# Stem Cells in Reproductive Tissues: From the Basics to Clinics

**DOI:** 10.1155/2013/357102

**Published:** 2013-04-07

**Authors:** Irma Virant-Klun, Thomas Skutella, Deepa Bhartiya, Xuan Jin

**Affiliations:** ^1^Reproductive Unit, Department of Obstetrics and Gynecology, University Medical Centre Ljubljana, Slajmerjeva 3, 1000 Ljubljana, Slovenia; ^2^Institute for Cell Biology and Anatomy, University of Heidelberg, Im Neuenheimer Feld 307, 69120 Heidelberg, Germany; ^3^Stem Cell Biology Department, National Institute for Research in Reproductive Health, Jehangir Merwanji Street, Parel, Mumbai 400 012, India; ^4^Centre for Assisted Reproduction, First Affiliated Hospital of Nanchang University, Nanchang, Jiangxi 330006, China

Twenty-one articles were accepted for publication in this special issue. All these contributions confirmed that human and animal reproductive tissues represent a very interesting and heterogeneous source of stem cells: from ovarian and testicular stem cells to embryonic (J. M. Campbell et al.; S.-J.Park et al.), trophoblast (M. Weber et al.), endometrial decidual (E. Rossignoli et al.), and umbilical cord stem cells, and induced pluripotent stem (iPS) cells. Moreover, recent research shows that, like in other adult organs and tissues, small embryonic-like stem cells (VSELs) are present in human reproductive tissues and may play an important role in the reproductive biology, as reviewed in the article “*Very small embryonic-like stem cells (VSELs): implications in reproductive biology*” by D. Bhartiya et al. 

There is more and more evidence that human spermatogonial stem cells are fact and not fiction. It is possible to short-term and long-term culture the human and animal spermatogonia (L. A. Martin and M. Seandel), and moreover, it is a time to critically stress the possibility of restoring fertility in sterile childhood cancer survivors by autotransplanting spermatogonial stem cells (R. B. Struijk et al.). An efficient clinical grade cryopreservation protocol for human testicular tissue and cells including spermatogonia is proposed by J. Pacchiarotti et al. In spite of the critical scientific debate if human testicular germ stem cells expressing a degree of pluripotency really exist, there is some further confirmation of their real existence. In the article “*In vitro culture-induced pluripotency of human spermatogonial stem cells,*” J. J. Lim et al. provided strong evidence that human germ stem cells from adult testis show similarities with embryonic stem cells being able to generate real teratomas after injection into SCID mice. Therefore, the natural plasticity of adult human spermatogonial stem cells could enable a clinical perspective in contrast to artificial iPS cells which are created by overexpression of transcription factors. In spite of that, iPS cells are a perfect model to study the *in vitro *spermatogenesis, as confirmed in the mouse model. The paper by P. Li et al. explored the differentiation potential of mouse iPS cells towards male germ cells; the expression of marker proteins, including MVH, CDH1, and SCP3, was remarkably increased, and mRNA expression of *Stra8*, *Odf2*, *Act*, and *Prm1 *was upregulated in iPS cells by retinoic acid or testosterone induction. Interestingly, there is also some new evidence that even mesenchymal stem cells may play an important role in restoration of fertility. In the very interesting research article “*Recovery of fertility in azoospermia rats after injection of adipose tissue derived mesenchymal stem cells: Sperm Generation*" by C. Cakici et al., the authors argue for the provocative idea of the *in vivo* transition and differentiation of adipose-derived mesenchymal stem cells into the direction of sperm after transplantation into the testes of sterilized rats. 

 Putative stem cells from adult human ovaries are an exciting and promising research subject which might lead to the clinical practice in the future. I. Virant-Klun et al. confirmed the development of oocyte-like cells from putative stem cells scraped from the ovarian surface epithelium of women with severe ovarian infertility-premature ovarian failure. These cells expressed several genes related to pluripotency and oocytes, as revealed by single-cell gene expression profiling, but were still more stem cells than real oocytes. Additionally, in the review paper “*Gene expression profiling of human oocytes developed and matured in vivo or in vitro*” by I. Virant-Klun et al., the authors present new knowledge about oocyte quality and oogenesis *in vivo* and *in vitro *in terms of gene expression profile, which might be introduced into clinical practice in the future. But the ovarian surface epithelium is far to be the only potential source of stem cells in adult human ovaries. K. C. Kossowska-Tomaszczuk and De Geyter reviewed the existence of stem cells in somatic compartments of the ovaries, including granulosa cells. In addition to the reproductive potential, there is still another important aspect of ovarian stem cells: the manifestation and potential new treatment of aggressive ovarian cancer. In their review article, J. Pasquier and A. Rafii exposed the importance of the microenvironment in ovarian cancer stem cell maintenance. Ovarian stem cells may be a new target for cancer therapy and more individualized treatment in the future, as proposed by Zhan et al.

Interestingly, there are also some other sources of stem cells which may be interesting for reproductive and regenerative medicine in the future. D. Zhang et al. were able to show in their article “*Estradiol synthesis and release in cultured female rat bone marrow stem cells*” that female bone-marrow-derived stem cells can synthesize and release estradiol and may contribute to autologous transplantation therapy for estrogen deficiency in reproductive medicine. Additionally, fetal stem cells might represent an important support to *in vitro* culturing and differentiation of stem cells. In the article by J. Xi et al., the authors describe an erythroid liquid culture system starting from cord blood-derived hematopoietic stem cells for the homogeneous erythroid cells cultured *in vitro*. The large number and purity of erythroid cells and red blood cells produced from cord blood make this method useful for fundamental research in erythroid development. J. Li and G. Lepski reviewed different sources of stem cells which can be used for spinal cord injury treatment in the future, including mesenchymal stem cells from human umbilical cord which have already been successfully differentiated into Schwann-like cells *in vitro* and grafted into the lesion sites of spinal cord injury rats; a partial recovery of motor function was reported. Last but not least, adipose-derived stem cells are an important future prospect for regenerative medicine. X. Zhu et al. successfully established a lentiviral vector encoding human hepatocyte growth factor (hHGF) and infected human adipose-derived stem cells. In this way they produced cells that overexpressed hHGF, which may provide a new strategy for the treatment of ischemic heart disease and other ischemic diseases in the future.

The human reproductive tissues provide an extremely interesting source of stem cells from both the basic and clinical views, which are more natural than iPS cells and also avoid several dilemma related to human embryonic stem cells. It was shown that the donation of surplus embryos for research is a worldwide problem. In the article of X. Jin et al. “*Patients' attitudes towards the surplus frozen embryos in China*,” it was found that 58.8% of the infertile couples included in the *in vitro* fertilization programme preferred to dispose surplus frozen embryos rather than donate them to research, mostly citing a lack of information and distrust in science as significant reasons for their decision. 

*Irma Virant-Klun* *Thomas Skutella* *Deepa Bhartiya* *Xuan Jin*

